# Lightweight Unet with depthwise separable convolution for skin lesion segmentation

**DOI:** 10.1038/s41598-025-16683-1

**Published:** 2025-10-02

**Authors:** Yong Li, Bosheng Hu, Xia Wang, Kun Liu

**Affiliations:** 1 College of Computer Science and Engineering, Northwest Normal University, Lanzhou, 730070 China; 2 College of Engineering, Northwest Normal University, Lanzhou, China; 3 Department of Pharmacy, People’s Hospital of Gansu Province, Lanzhou, China

**Keywords:** Skin segmentation, Lightweight model, Depthwise separable convolution, Skin diseases, Image processing

## Abstract

Accurate segmentation of skin lesions is crucial for the early diagnosis of skin diseases, as clear lesion boundaries facilitate the comprehensive extraction of lesion features. However, in practical applications, the contours or boundaries of lesion areas in medical images often appear blurred and lose detail due to factors such as lighting conditions, imaging equipment, skin color differences, and physician experience. Meanwhile, existing deep learning models are often large in structure and computationally expensive, making them unsuitable for clinical environments that require efficiency and ease of deployment.To address these issues, this paper proposes a lightweight skin lesion segmentation model, LMSAUnet. Based on the traditional UNet architecture, this model removes standard convolution modules and introduces the ECDF module, which combines deep separable convolution and attention mechanisms. This significantly reduces the number of parameters and computational complexity while improving segmentation performance.Experimental results demonstrate that LMSAUnet outperforms multiple state-of-the-art segmentation models on several skin lesion datasets, significantly reducing computational resource consumption while exhibiting stronger generalization capabilities. The findings validate the model’s potential for efficiently and accurately performing skin lesion segmentation tasks under resource-constrained conditions, offering promising clinical application prospects.

**Supplementary Information:**

The online version contains supplementary material available at 10.1038/s41598-025-16683-1.

## Introduction

According to the World Health Organization, there are more than 3,000 different skin diseases that affect not only the skin, but also mucous membranes, nails and hair. These diseases collectively affect over 3 billion individuals across all age groups globally, constituting the ​fourth leading cause of non-fatal disease burden​​ and the ​​seventh leading cause of years lived with disability (YLDs)​​ worldwide. Malignant melanoma, in particular, is responsible for about 73% of skin cancer-related deaths^1^. Studies have found that five-year survival rates can be increased to 95% if detected early, but only 20% when diagnosed at an advanced stage, highlighting the urgent need for early and accurate diagnosis.

**Fig. 1:**
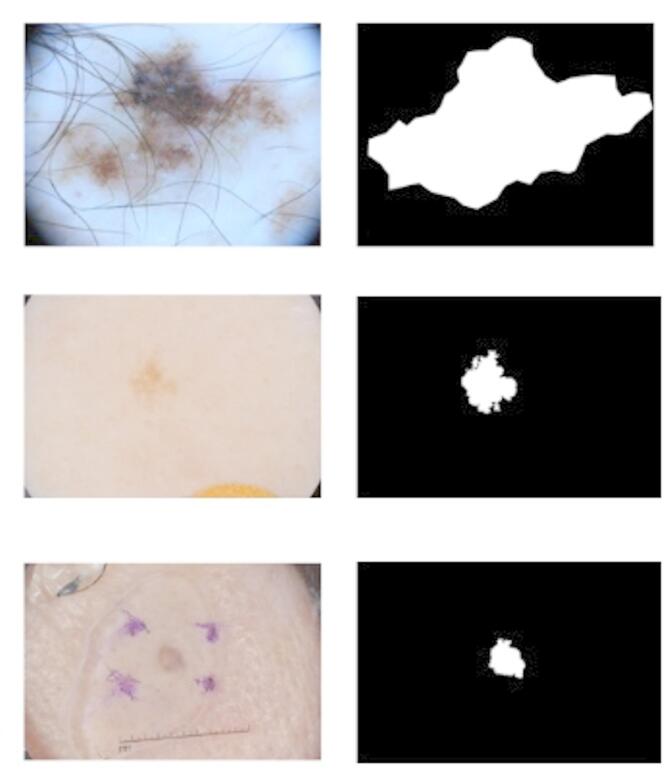
Left: original lesion image. Right: corresponding segmentation result.

However, the heterogeneity of skin lesions (e.g., variable color and shape) and external disturbances (hair obscuration, uneven illumination, etc.) make manual recognition difficult (Fig. 1). Although tools such as dermoscopy can be used that can aid in observation, it still relies on the subjective judgment of the physician. Therefore,​ the development of automated image segmentation techniques​ to overcome problems such as low contrast and blurred boundaries has become the key to improving diagnostic efficiency and accuracy.

Early traditional medical image segmentation methods (e.g., active contour modeling^2^ have been gradually replaced by deep learning methods due to their reliance on manual feature design, which makes it difficult to process lesion images in complex situations. The current mainstream scheme is represented by U-Net^3^whose encoder-decoder structure fuses multi-scale features through jump connections, making a breakthrough in skin lesion segmentation. Subsequent TransUNet^4^introduced Transformer^5^ module to enhance the global modeling capability, but it suffers from the defects of high computational complexity and poor generalization to small samples^6^. To address these problems, lightweight improvement schemes such as ConvNeXt proposed^7^reduce the number of parameters by optimizing the convolutional kernel design, and PP-LiteSeg^8^enhances the inference efficiency by using pyramidal pooling. However, these methods still face triple challenges in skin lesion scenarios:

1. The contradiction between accuracy and computational efficiency: the number of parameters of existing models usually exceeds 25M^9^ when keeping the Dice coefficient greater than 0.85, resulting in GPU inference latency greater than 50ms, which is difficult to meet clinical real-time demands (e.g., dermatoscopic dynamics examination).
2. Inadequate cross-center domain adaptation​ :In ISIC 2020 cross-center testing, the model’s performance degradation from the Derm7pt to PH2 dataset was up to 18.7%^10^, mainly stemming from device imaging differences (e.g., polarized light and unpolarized dermatoscopy).
3. Poor adaptation to dynamic examination scenarios: in real-time dermoscopy, the model is significantly less robust to hair occlusion and bubble artifacts. The segmentation Dice coefficient decreases by 23.5% on average when the hair coverage in the image is > 15%^11^. This type of noise interference in dynamic environments is prevalent in real clinical situations, and if the model does not possess sufficient robustness, it will severely limit its clinical application value.

To address the dual requirements of efficiency and practicality in the automatic segmentation of clinical skin lesions, this paper proposes a novel lightweight segmentation model, LMSAUnet. The model integrates deep separable convolutions and spatial-channel attention mechanisms, achieving accurate segmentation performance and superior cross-domain generalization ability on multiple public datasets with less than 0.4 M parameters.Additionally, the ECDF module we designed effectively integrates multi-scale channel features and enhances the response to critical lesion regions through a dynamic channel weighting mechanism. Due to its high efficiency, low resource consumption, and excellent transferability while maintaining high performance, LMSAUnet has the potential for deployment and application in resource-constrained clinical environments, offering a promising technical foundation for skin lesion auxiliary diagnosis.

The main contributions of this paper are as follows:

1. The lightweight skin lesion image segmentation model is proposed LMSAUnet to solve the problem of the difficult balance between the number of parameters and floating point of operations and model performance. experimentally It is proved that: by taking advantage of the characteristics of the high channel redundancy and introducing the depth separable convolution and channel attention mechanism, the model parameters and training burden are reduced dramatically, and in the case of the number of parameters is less than 0. 4 M, the Dice coefficient is improved to 0. 8792, which completely outperforms the performance of the benchmark model.
2. Keeping the model lightweight not only reduces resource requirements but also enhances cross-domain capabilities across various devices, environments, and regions, addressing the issue of high accuracy on training sets but insufficient generalization ability in actual applications. Experiments demonstrate that the proposed LMSAUnet still outperforms current mainstream models on untrained external datasets.
3. A multi-scale feature extraction and attention guidance mechanism is introduced into the model structure to effectively strengthen the saliency of the lesion region, and at the same time suppress the influence of background interference such as hairs and air bubbles. The discriminative ability of the model for boundary blurring and low-contrast regions is improved by means of feature guidance. Experiments demonstrate that the model still maintains high segmentation accuracy in datasets with high hair occlusion rate or significant background noise interference, showing good robustness and clinical adaptability.
4. In this study, a modular feature extraction structure, ECDF, is designed to incorporate multisensory field context information to enhance feature expression capability. The module is characterized by low structural coupling and low computational overhead, which facilitates flexible embedding into existing backbone networks to enhance segmentation performance. Experiments show that integrating the ECDF module into the mainstream model can significantly reduce the computational effort while maintaining or even exceeding the segmentation effect of the original module, demonstrating good generalization and scalability.

## Related work

Skin lesion segmentation algorithms can be broadly categorized into two main types. The first category comprises classical unsupervised algorithms, such as clustering algorithms and threshold segmentation and edge detection based on image statistical features. The second category consists of neural networks in deep learning.

Unsupervised algorithms are a class of image processing techniques that focus on identifying and extracting inherent low-level features in images. They are widely used in image segmentation, particularly for datasets without labels. These algorithms typically rely on low-level image features such as pixel values, color, and texture, making them computationally efficient and suitable for resource-constrained or real-time scenarios.

Clustering algorithms are one of the most common unsupervised segmentation techniques. K-means^12^ clustering groups pixels in an image so that each pixel belongs to the nearest cluster center. While this method is simple and computationally efficient, it is sensitive to initial cluster centers and may be affected when handling complex boundaries. In contrast, C-means^13^ clustering (fuzzy C-means clustering) allows pixels to belong to multiple clusters and assigns different degrees of membership, making it suitable for handling blurry or uncertain boundaries.

Threshold-based segmentation methods^14^ are a classic and efficient technique. They analyze the grayscale or color distribution of an image to set a threshold for distinguishing lesion regions. Global threshold segmentation methods select thresholds based on the overall grayscale histogram but are susceptible to background changes and lighting conditions. Local threshold segmentation, on the other hand, sets different thresholds for different regions, enhancing adaptability to complex backgrounds or images with uneven lighting.

Edge extraction methods perform well when there is strong contrast between the lesion area and the background. Canny edge detection^15^ detects image edges through gradient changes and is suitable for lesions with distinct boundaries. The Sobel operator extracts horizontal and vertical edges through convolution operations, offering a simple and effective solution suitable for real-time applications.

Although unsupervised methods are computationally efficient, they often suffer from low accuracy, sensitivity to noise, and difficulty in handling complex, blurry, or irregular images. Most methods lack adaptive capabilities for complex images, resulting in recognition accuracy significantly lower than modern deep learning networks^16^.

In recent years, deep learning has become the mainstream approach for image segmentation, detection, and recognition in computer vision. With enhanced computational power, deep learning has made significant strides, leading to new developments in medical image processing, most notably the fully convolutional network (FCN). FCN^17^ achieves this by replacing the fully connected layers in traditional CNNs with convolutional layers, enabling the network to make pixel-wise predictions within images. This is crucial for tasks such as medical image segmentation. The FCN architecture has demonstrated high effectiveness in extracting spatial hierarchical structures and contextual features from medical scan images, particularly in CT and MRI images, which often exhibit complex patterns and intricate structures. Building on the success of FCN, further innovations have emerged to address some limitations in medical image segmentation. A notable advancement is the U-Net architecture, which retains high-resolution spatial information through the introduction of skip connections, thereby improving the accuracy of pixel-level predictions. In addition, Transformer architectures such as Vision Transformer^18^ and Swin Transformer^19^ have been introduced into the field of medical image segmentation. Compared to traditional convolutional neural networks, the attention mechanism is adept at capturing long-range dependencies in images, which is particularly effective for complex medical image segmentation tasks.Therefore, a large number of models combining convolutional neural networks and attention mechanisms have been proposed at this stage, such as TESL-Net^20^ and AD-Net^21^.

Furthermore, with the widespread application of deep learning in medical image segmentation, researchers have begun to focus on developing lightweight segmentation models to meet the high demands for computational resources and real-time performance in clinical environments^22^. While traditional deep learning models perform well, their large parameter counts and high computational requirements often make them difficult to run efficiently on resource-constrained devices. As a result, lightweight convolutional neural networks (CNNs) and other efficient architectures have become a research hotspot. Therefore, the researchers proposed the EGE-Unet^23^ model with an improved attention mechanism, the UCM-Net^24^ model consisting only of linear layers and convolutional layers, and the MUCM-Net^25^ model consisting of a state space model and convolutional layers, which significantly reduced the number of parameters.

Additionally, researchers have proposed lightweight segmentation methods based on distillation learning^26,27^. By transferring knowledge from a large-scale, high-performance teacher network to a smaller student network, distillation learning enables lightweight models to achieve significant reductions in parameters and computational complexity while maintaining high segmentation accuracy. This method significantly improves model efficiency in medical image segmentation tasks such as skin lesion detection, particularly when deployed on edge computing devices and mobile devices.

In addition to model structure optimization, lightweight training techniques are also evolving. Through pruning^28^ and quantization^29^ techniques, researchers can reduce redundant parameters in the network while maintaining model accuracy, further improving model inference speed and deployment efficiency. These techniques can significantly enhance the response speed of segmentation models in practical applications, meeting the demands of real-time clinical analysis.

In summary, as the demand for medical image segmentation continues to grow, lightweight models are increasingly becoming an important research direction. By adopting techniques such as deep separable convolutions, distillation learning, model pruning, and quantization, researchers have not only improved segmentation accuracy but also ensured the model’s efficiency and usability in environments with limited computational resources. These lightweight segmentation models will open up new possibilities for intelligent diagnosis and real-time decision-making in clinical practice.

**Fig. 2:**
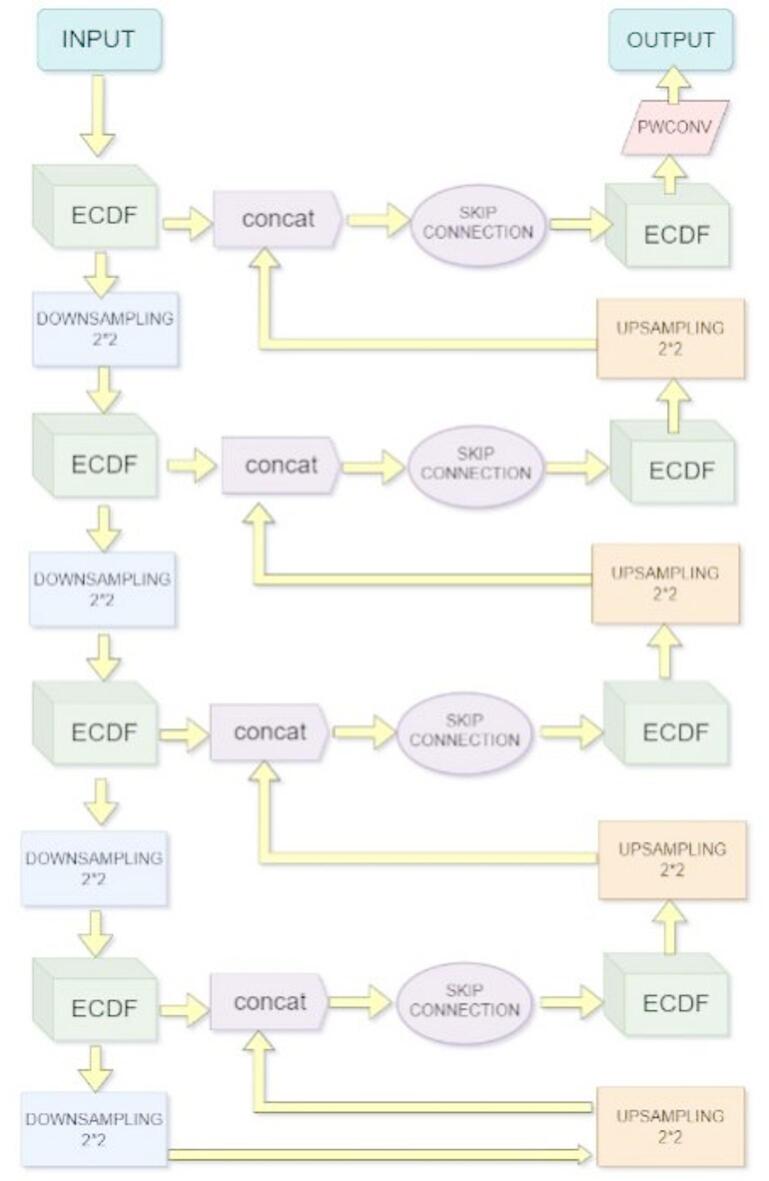
Framework of LMSAUnet: Encoder with interleaved ECDF modules and downsampling, and a symmetrically mirrored decoder incorporating skip connections for refined segmentation detail preservation.

## Method

### LMSAUnet

As shown in Fig. 2, this paper proposes a lightweight variant model based on the UNet architecture. The model retains the classical U-shaped encoding-decoding structure, but makes key improvements to address the parameter count explosion problem of traditional UNet.

**Table 1:** Feature map size and channel changes across different blocks of the proposed LMSAUnet architecture.

Blocks	Output feature map size	Number of input channels	Number of output channels
ECDF BLOCK 1	H * W	3	64
DOWNSAMPLING 1	H/2 * W/2	64	64
ECDF BLOCK 2	H/2 * W/2	64	128
DOWNSAMPLING 2	H/4 * W/4	128	128
ECDF BLOCK 3	H/4 * W/4	128	256
DOWNSAMPLING 3	H/8 * W/8	256	256
ECDF BLOCK 4	H/8 * W/8	256	512
DOWNSAMPLING 4	H/16 * W/16	512	512
UPSAMPLING 1	H/8 * W/8	512	512
ECDF BLOCK 5	H/8 * W/8	1024	512
UPSAMPLING 2	H/4 * W/4	512	512
ECDF BLOCK 6	H/4 * W/4	768	256
UPSAMPLING 3	H/2 * W/2	256	256
ECDF BLOCK 7	H/2 * W/2	384	128
UPSAMPLING 4	H * W	128	128
ECDF BLOCK 8	H * W	192	64
PWCONV	H * W	64	2

Each block illustrates the corresponding output resolution (H × W), input channels, and output channels at that stage.

The encoder stages in the original Unet achieve feature extraction by two 3 × 3 convolutions (the number of channels grows by an exponential power of 2) and one downsampling, while the decoder achieves upsampling by inverse convolution in conjunction with convolution operations to compress the channels. Its parametric quantity for only one convolution operation is shown in eq. (0) where K is the convolution kernel size:

0 $$\:\begin{array}{c}Params={C}_{in}\times\:{C}_{out}\times\:K\times\:K\end{array}$$

At deeper layers of the network (e.g., number of channels from 512 to 1024), this design will result in a high number of parameters for only the convolutional operation of the layer of 9.43 M, resulting in significant storage and computational overhead.

For this reason, this paper proposes the ECDF module to replace the traditional two-time convolution in encoders and decoders. While significantly reducing the number of parameters, the ECDF module can still effectively accomplish the feature extraction and channel compression tasks, and its specific structure will be described in detail in the next subsection.

The encoder part of the model adopts a four-stage incremental structure, with each stage consisting of an ECDF module and a 2 × 2 downsampling operation. The main processing flow of each encoder level is as follows:

1. ECDF module: used to replace the two 3 × 3 convolution operations in each stage in traditional UNet, significantly reducing the number of parameters while retaining strong feature extraction capability.
2. Downsampling operation (2 × 2): downsampling of the feature map by average pooling or maximum pooling with a step size of 2 reduces the resolution and increases the receptive field.

Table 1 shows the changes in the number of feature image channels, as the layers deepen, the number of channels of the feature map gradually increases, while the spatial size decreases layer by layer, realizing the gradual refinement from shallow local features to deep global semantic features. The features of each layer are simultaneously retained in the decoder through skip connections for subsequent high-resolution semantic information recovery.

In the decoder section, the model employs a four-stage upsampling structure, symmetrical to the encoder. Each level of decoding operation consists of the following steps:

1. Bicubic interpolation up-sampling: Used to up-sample low-resolution feature maps to a higher resolution, compared with the traditional Unet deconvolution operation, bicubic interpolation does not need to learn the parameters, which significantly reduces the memory usage.
2. Feature map splicing : The up-sampled feature map is spliced with the jump-connected features of the corresponding layer in the encoder in channel dimension to enhance the spatial detail information.
3. ECDF module processing: the spliced feature maps are input to the ECDF module to further fuse the local and global information to enhance the semantic recovery capability.

### ECDF

**Fig. 3:**
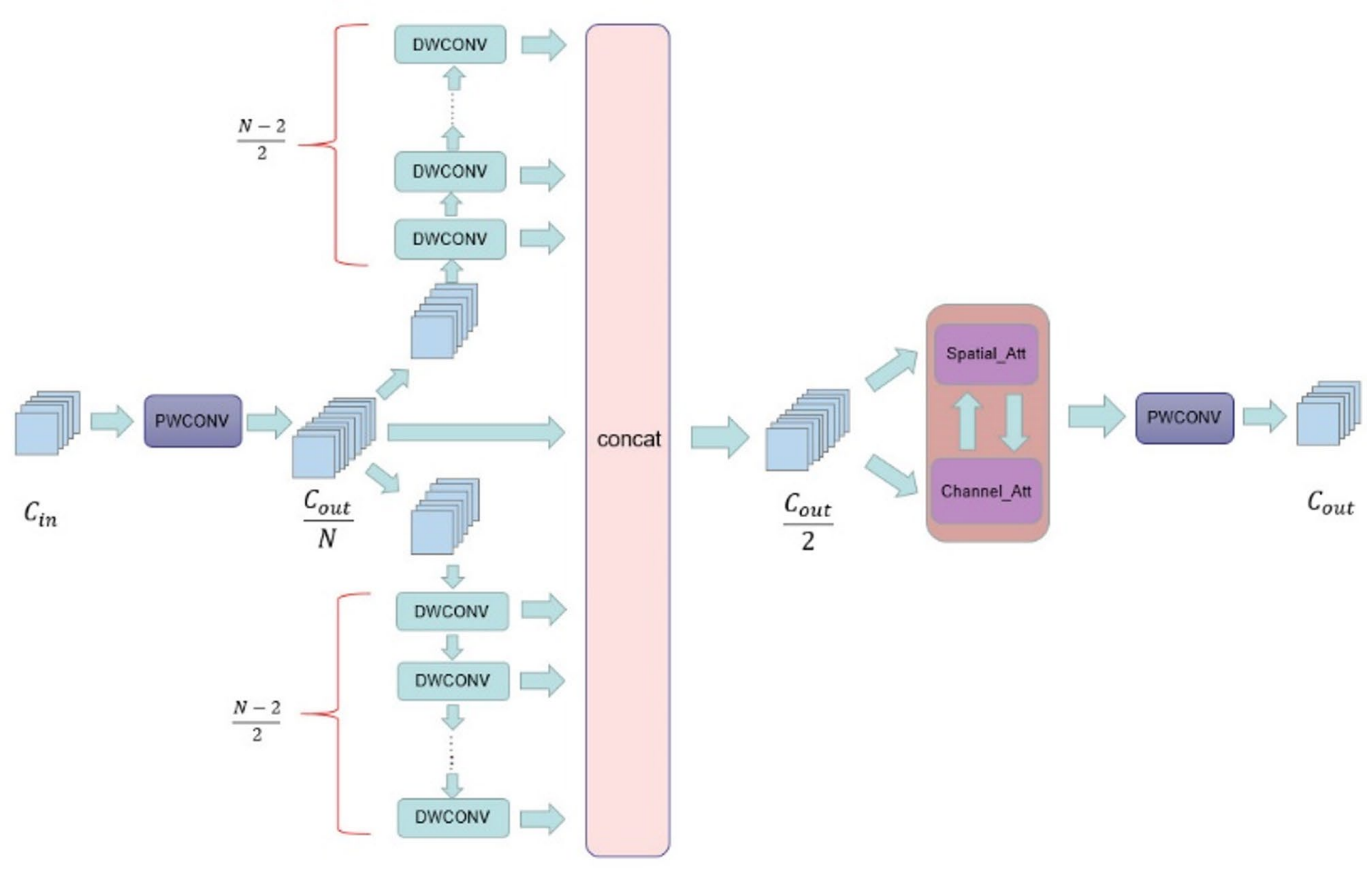
The structure of the ECDF module.

To cope with the problems of exploding number of parameters and high memory occupation in traditional UNet, this paper designs a lightweight feature extraction unit, ECDF module. This module is used to replace the two 3 × 3 convolutional structures at each stage in the original UNet to achieve similar or better feature extraction capability with higher computational efficiency, The module structure of ECDF is shown in Fig. 3.

The ECDF module references the design of the Xception network and uses depth-separable convolution as the main part of the feature extraction. Deep separable convolution has two operations: deep convolution, in which each convolution kernel acts on only one input channel, extracts spatial features within the channel, and generates a new feature map with constant channel dimensions. Point-by-point convolution, in which a 1 × 1 convolution kernel is used to fuse in the channel dimension, enabling the integration of information between channels^30^.

However, unlike the Xception network which enhances its expressive capability by expanding the number of channels in the middle layer, the ECDF module takes full advantage of the existence of redundancy in the existing feature map channels while keeping the number of channels unchanged^31^and by accomplishing efficient depth-separable convolutional operations, incorporates multiple scales and semantic information. Meanwhile, to enhance the model’s sensitivity channelsto key regions and, the ECDF module integrates a spatial-channel attention mechanism. The mechanism refers to the design idea of CBAM^32^. In the implementation, the parallel structure of channel-attention and spatial-attention are computed independently, and the 1D convolution is instead of the traditional fully-connected layerused, in order to further alleviate the parameter burden of the attention mechanism and at the same time to enhance the model’s responsiveness to the salient regions. The ablation experiments confirm that the attention mechanism has a significant effect on the model performance, and the specific experimental results are shown in Table 6.

In order to reduce the computational overhead of the middle layer, this study introduces a channel compression factor N to control the number of channels of the middle layer feature map. Specifically, let the input feature map be$\:\:X\in\:{R}^{{C}_{in}\times\:H\times\:W}$, the output feature map be $\:Y\in\:{R}^{{C}_{out}\times\:H\times\:W}$, and the number of channels in the middle layer be$\:\:{C}_{mid}={C}_{out}/N$, the compression factor N satisfies$\:\:N={2}^{K}$, where *K* is a non-negative integer, the larger the factor is, the more obvious the compression is, and the smaller the computational overhead is, but it may also result in the loss of feature information. Therefore, we systematically evaluated different N taking values in our experiments (see Ablation experiments).

Han et al. pointed out that there are a large number of redundant channels in the feature map^33^and ECDF considers to deal with feature redundancy in a low-cost way, as shown in Fig. 3, which first compresses the feature dimensions by point-by-point convolution to $\:{X}^{{\prime\:}}\in\:{R}^{{C}_{\frac{out}{N}\times\:H\times\:W}}$, the channels of the compressed feature map are divided into two parts, each of occupies$\:\:\frac{{C}_{out}}{2N}$ which channels performs (*N* − 2)/2 deep convolution operations on these two parts, and saves the results of each convolution. Finally, the results of a total of (*N* − 2) deep convolutions are spliced with the initial point-by-point convolved feature maps along the channel dimensions to obtain the feature maps of with the size of$\:\:\frac{out}{2}\times\:H\times\:W$, at this stage, the incorporation of multi-level semantic information within a singular feature map has been accomplished, as in Eq. (1). The feature map acquires spatial and channel dimensional attention information in parallel at next stage and adaptively adjusts the importance of the two attention mechanisms with learnable weights. The fused attentional weights are then applied to the original feature map to enhance the model’s ability to perceive and represent locally salient features. To fully utilize the information from different receptive fields and channels, an additional point-by-point convolution is introduced at the end of the ECDF module to adjust the dimension of the final feature maps to $\:Z\in\:{R}^{{C}_{out}\times\:H\times\:W}$ in order to fuse the channel features and output them to the subsequent modules.

1 $$\:\begin{array}{c}(N-2)\frac{{C}_{out}}{2N}+\frac{{C}_{out}}{N}=\frac{\left(N-2\right){C}_{out}+2{C}_{out}}{2N}=\frac{{C}_{out}}{2}\end{array}$$

#### Point-by-point Convolution

**Fig. 4:**
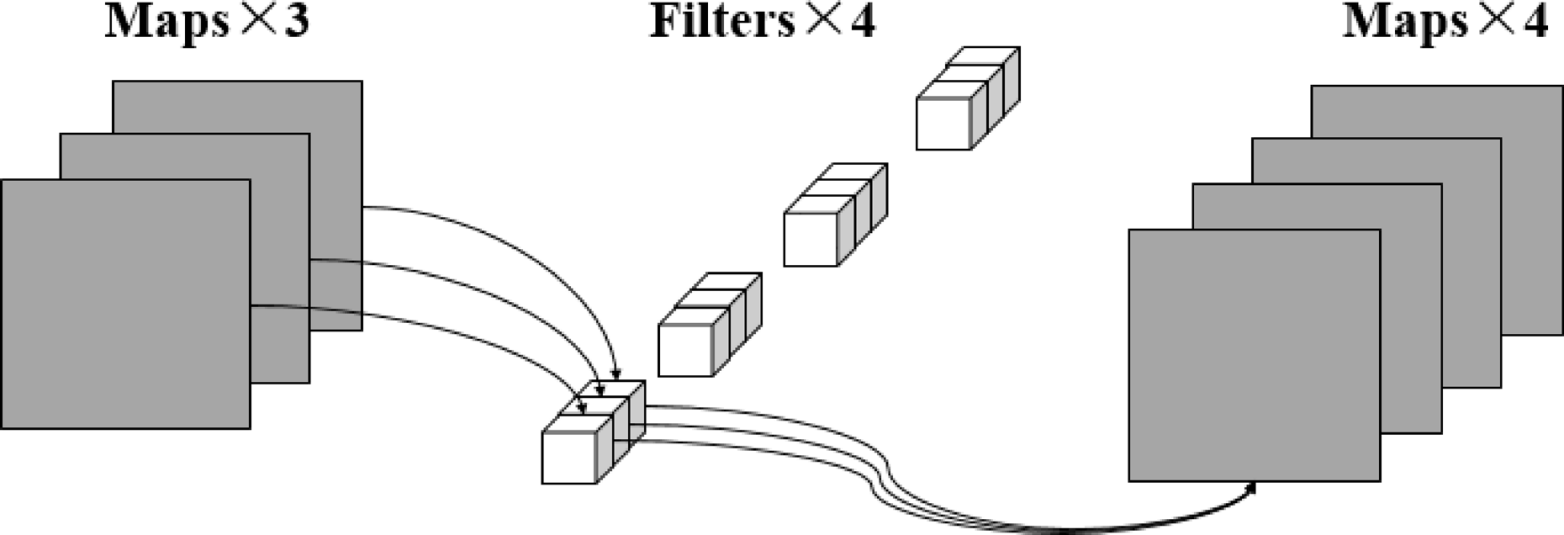
Pointwise convolution: 3 input channels→ 4 output channels via four 1 × 1 × 3 kernels.

As in Fig. 4, point-by-point convolution refers to the use of a convolution kernel of size 1 × 1 to linearly transform each spatial location of the input feature map, the mathematical expression of Eq. (3) Its main role is to adjust the channel dimension without changing the spatial size of the feature map. Let the input feature map be $\:X\in\:{R}^{{C}_{in}\times\:H\times\:W}$, the weight of point-by-point convolution is$\:\:W\in\:{R}^{{C}_{out}\times\:{C}_{in}\times\:1\times\:1}$, then the output feature map is$\:{\:Y}_{pw}\in\:{R}^{{C}_{out}\times\:H\times\:W}$, and the number of its parameters is calculated as equation (2):

2 $$\:\begin{array}{c}\:\:Params={C}_{in}\times\:{C}_{out}\end{array}$$

In contrast, the number of parameters of conventional convolution is Eq. (0), so the number of parameters of point-by-point convolution is about $\:\:\frac{1}{{K}^{2}}$ of conventional convolution, which significantly reduces the model complexity and computational overhead, and the point-by-point convolution process can be expressed by Eq. 3.

Nevertheless, the limitations of point-by-point convolution are more obvious:

1. Point-by-point convolution lacks the ability to model local spatial information because a 1 × 1 convolution kernel cannot capture the spatial relationships between pixels;
2. When there are complex dependency structures between channels, point-by-point convolution may not adequately model these relationships.

As in Fig. 4:

3 $$\:\begin{array}{c}{\:\:\:\:\:\:\:\:\:\:\:Y}_{pw}(h,w,{C}_{out})=\sum\limits_{{C}_{in}=1}^{{C}_{in}}{W}_{pw}\left({C}_{out},{C}_{in},\text{1,1}\right)\cdot\:X\left(h,w,{C}_{in}\right)\end{array}$$

#### Deep Convolution

**Fig. 5:**
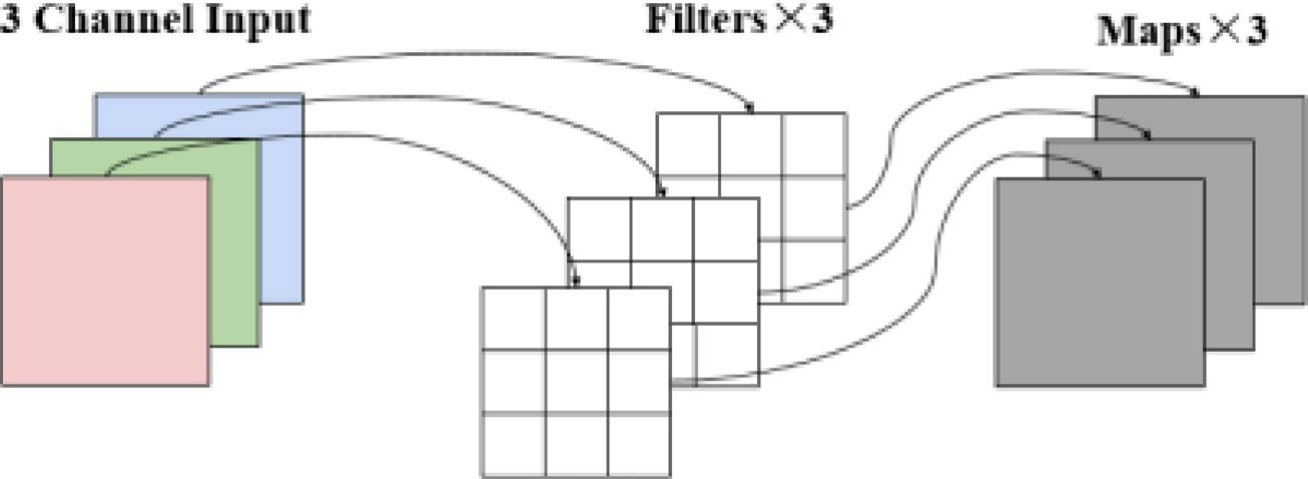
Depthwise convolution example: Input (3 channels)→ 3 kernels (1 per channel)→ Output (3 channels).

Deep convolution is an efficient spatial feature extraction method characterized by the fact that each input channel corresponds to an independent convolution kernel, which performs spatial convolution operations individually without involving inter-channel fusion, as shown in Fig. 5.

Let the input feature map be $\:X\in\:{R}^{Cin\times\:H\times\:W}$ and the convolution kernel be $\:{W}_{dw}\in\:{R}^{C\times\:K\times\:K}$, then the output feature map$\:\:{Y}_{dw}\in\:{R}^{C\times\:H\times\:{W}^{{\prime\:}}}$ is given by (4):

4 $$\:\begin{array}{c}{\:\:\:\:\:\:\:Y}_{dw}^{c,h,w}=\sum\limits_{i=0}^{K-1}\sum\limits_{j=0}^{K-1}{W}_{dw}^{c,i,j}\cdot\:{X}^{c,h+i,w+j}\end{array}$$

This operation can effectively extract local spatial features within a single channel and uses the cross-channel parameter redundancy in traditional convolution. Therefore, deep convolution is often paired with point-by-point convolution to alleviate the problem of poor local region perception by point-by-point convolution.

#### Spatial channel attention

The spatial attention mechanism is a technique that enhances the model’s ability to perceive critical areas by dynamically adjusting the importance of different spatial locations in the input feature map.

The specific implementation process is as follows: firstly, pooling operation across channels is performed on the input feature map to compress the channel dimension and retain the spatial information. Usually, global maximum pooling and global average pooling are adopted to take the maximum and average values for each spatial location in the channel dimension, respectively, to generate a feature map with the shape of $\:X\in\:{R}^{2\times\:H\times\:W}$^34^.Subsequently, the compressed feature map is fed into a convolutional layer to generate a spatial attention weight map of shape$\:\:{W\in\:R}^{1\times\:H\times\:W}$. The weight map is normalized by a Sigmoid activation function, and the values of each spatial position are mapped to the interval (0, 1) to reflect the importance of the corresponding position. Finally, the spatial attention map is multiplied by the original feature map to achieve the feature enhancement of the significant regions in the spatial dimension, which helps to improve the discriminative ability and generalization performance of the model.

Let the input feature map be $\:\text{X}\in\:{R}^{C\times\:H\times\:W}$, first perform maximum pooling and average pooling operations on each spatial location (h, w) in the channel dimension to obtain two single-channel feature maps:

5 $$\:\begin{array}{c}{F}_{max}(h,w)={max}_{c}X(c,h,w)\:,{F}_{avg}(h,w)=\frac{1}{C}\sum\limits_{c=1}^{C}X\left(c,h,w\right)\end{array}$$

The two are spliced in the channel dimension to obtain a compressed two-channel feature map:

6 $$\:\:{\:\:\:\:\:\:\:\:\:\:\:\:F}_{concat}=\text{C}\text{o}\text{n}\text{c}\text{a}\text{t}\left({F}_{max},{F}_{avg}\right),{F}_{concat}\in\:{R}^{2\times\:H\times\:W}$$

Next, a convolution operation is performed on $\:{F}_{concat}$ using the convolution kernel $\:\text{W}\in\:{R}^{k\times\:k\times\:2\times\:1}$ to generate the spatial attention mapping:

7 $$\:G=W\cdot\:{F}_{concat}\:,\text{Y}\left(h,w\right)={\upsigma\:}\left(\text{G}\left(h,w\right)\right)\in\:\left[\text{0,1}\right]$$

Where $\:{\upsigma\:}(\cdot\:)$ is the sigmoid function used to normalize the attention values to the interval [0, 1]. Ultimately, Spatial attention weight map completed.

The channel attention mechanism is a feature recalibration technique commonly used in deep learning, and its core idea is to enhance the response of key channels, suppress redundant or noisy channels, and dynamically assign weights to optimize the expressiveness of each channel in the feature map by automatically learning the degree of contribution of different channels to the final task through the network.

The implementation of this mechanism is similar to spatial attention, and in this paper, we refer to the design of ECA Block^35^which introduces a lightweight convolutional structure to reduce the computational overhead. The specific process is as follows:

Let the input feature map be $\:X\in\:{R}^{C\times\:H\times\:W}$, and first perform global average pooling for each channel to obtain a channel-level statistics vector:

8 $$\:\:\:\:\:\:\:\:\:\:\:\:\:\:\:\:\:\:{S}_{c}=\frac{1}{H\times\:W}\sum\:_{h=1}^{H}{\sum\:}_{w=1}^{W}{X}_{c,h,w},\:\forall\:c\in\:\left\{\left.\text{1,2},\dots\:,\text{C}\right\}\right.$$

The output after pooling is $\:S\in\:{R}^{C\times\:1\times\:1}$ .

Subsequently, in order to model the inter-channel dependencies and control the computational complexity, the channel vectors are processed using an adaptively sized one-dimensional convolution kernel. The convolution kernel size *K* is computed adaptively based on the number of channels *C*, as defined in Eq. (9) of :

9 $$\:\:\:\:\:\:\:k={\left\lfloor\frac{{\text{log}}_{2}C}{\gamma\:}+b\right\rfloor}_{odd}$$

where γ and b are hyperparameters, and $\:{\lfloor\:\cdot\:\rfloor\:}_{odd}$ denotes rounding down to the nearest odd number.

The vector *S* is adjusted to $\:S\in\:{R}^{1*c}$ and then convolved with a one-dimensional convolution kernel $\:{W}_{k}\in\:{R}^{1*k}$ and normalized by the Sigmoid function to obtain the channel weights:

10 $$\:\:\:\:\:\:\:\:\:\:\:\:\:\:\:\:\:\:\:\:\:\:\:\:\:\:\:Y={\upsigma\:}({W}_{k}*S)\in\:{R}^{C\times\:1}\:$$

At this point, we have obtained the channel attention weights.

**Fig. 6:**
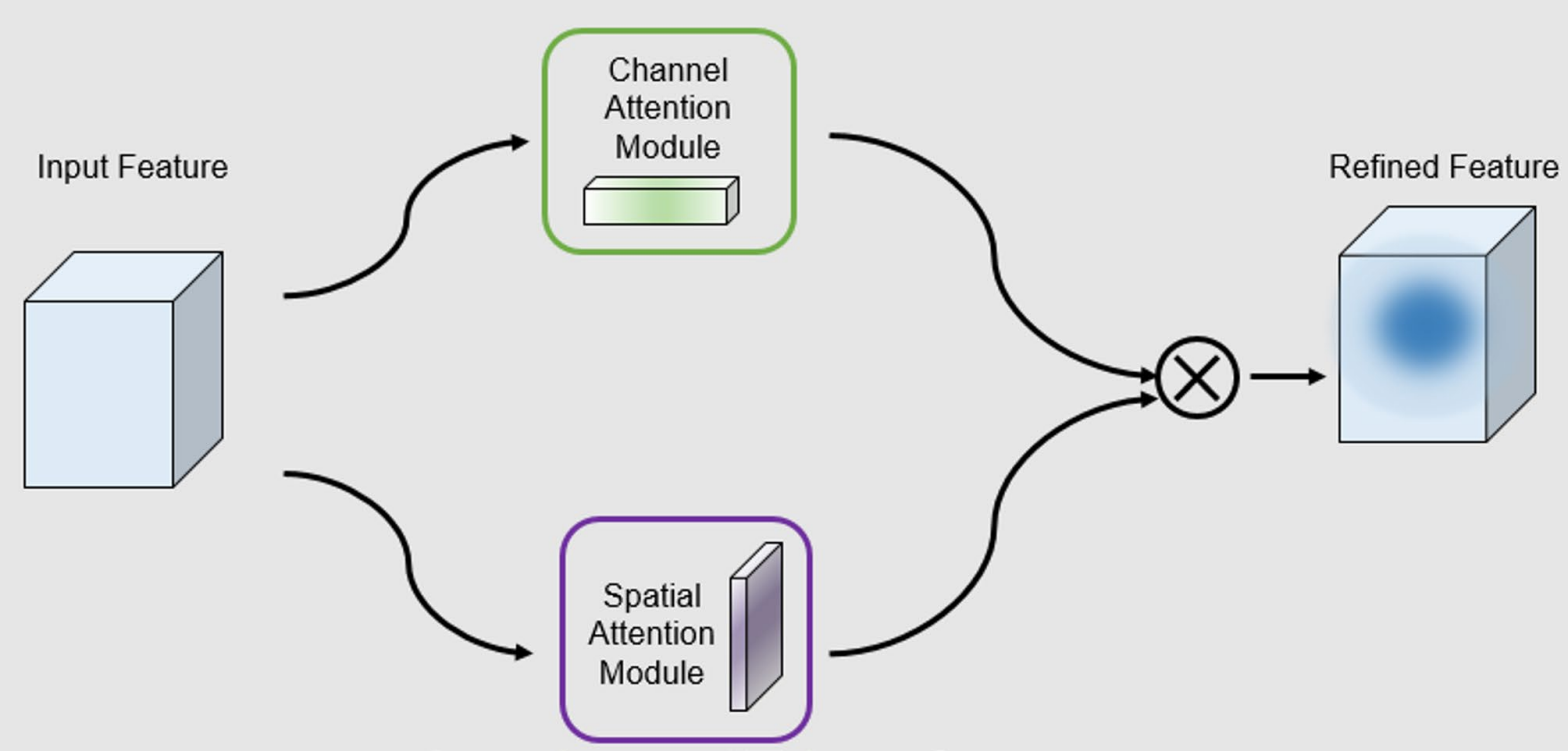
Channel and spatial attention weights (scaled by α/β) are applied to the input feature map for enhanced feature representation.

Finally, channel attention and spatial attention are fused in parallel, and both are computed separately and then integrated by weighted summation, as shown in Fig. 6, so as to take into account both spatial and channel important information while keeping the structure lightweight.

The form of integration is as follows:

11 $$\:{X}^{{\prime\:}}=X\cdot\:\left({\upalpha\:}\cdot\:{Y}_{channelWeight}+{\upbeta\:}\cdot\:{Y}_{spaceWeight}\right),{X}^{{\prime\:}}\in\:{R}^{C\times\:H\times\:W}$$

where $\:{Y}_{channelWeight}$ and $\:{Y}_{spaceWeight}$ denote the feature maps enhanced by channel attention and spatial attention, respectively, and α and β are learnable parameters used to control the influence weights of the two attentional mechanisms in the fusion process.

## Results

### Datasets

The dataset used in this study is derived from publicly available data provided by the International Skin Imaging Collaboration (ISIC), which has been used to advance computer vision-based research on automated diagnosis of skin lesions, and has been particularly instrumental in the early detection of malignant lesions such as melanoma. The dataset comes from multiple medical institutions, covers a variety of dermoscopic devices (e.g., Heine, Canfield) with different imaging conditions (light, focal length, etc.), involves patient populations with different skin colors, ages, and lesion sites, and dataset has a good diversity and cross-domain coverageincludes common types of skin lesions, such as melanoma, pigmented nevus, basal cell carcinoma, etc. The. Therefore, it has good diversity and cross-domain representativeness, and is an ideal data source for assessing the generalization ability of the model. The dataset ISIC has become the authoritative evaluation benchmark in the field of dermatological segmentation and classification.

The PH² dataset was constructed by the Skin Imaging Research Group in Porto, Portugal, focusing on early detection studies of cutaneous melanoma. The dataset contains 200 high-resolution images of skin lesions, each labeled by a professional dermatologist to ensure accuracy and of the labelsmedical credibility, with segmentation masks of the lesion areas and clinical diagnosis.

In this paper, skin lesion segmentation datasets from ISIC 2016^36^, 2017^37^ and 2018^37^ years were selected as the main training and testing datasets. PH²^38^ dataset was used as an external dataset to verify its generalization. We followed the officially provided segmentation for the experiments, as detailed in Table 2:

**Table 2:** Partitioning of the datasets used in this study, including three public datasets and one internal dataset.

Data set	Training set	Test set	Validation set
ISIC2016	900	267	112
ISIC2017	2000	150	600
ISIC2018	2594	1000	100
PH²	–	200	–

### Realization detials

The training of the model was carried out on an NVIDIA A30 GPU based on 30GB of graphics memory, implemented using the PyTorch framework. The optimizer is AdamW with the momentum decay factor set to $\:{1\times\:10}^{-5}$. The number of training rounds is capped at 200, and an early-stop strategy is introduced: if the loss value of on the validation set does not decrease within 20 consecutive cycles, the training is terminated early to prevent overfitting. During the whole training process, the batch size is set to 8 to balance the training speed and memory consumption efficiency.

In terms of the learning rate strategy, a cosine annealing learning rate scheduler is used, with the initial learning rate set to $\:{1\times\:10}^{-6}$, and the learning rate dynamically changes with a cosine waveform during the training process in order to increase the convergence speed of the model and to have a better chance of jumping out of the local optimum. We resize the images to 256 × 256 to fit the model’s input.As for the loss function, in order to optimize the pixel-level segmentation accuracy, crossentropy is used in combination with the IoU loss function to construct a joint loss function for supervised training of the model, and its loss function expression is as follows.

Let $\:\widehat{y}\left(h,w\right)\in\:\left[\text{0,1}\right]$ be the predicted value after sigmoid,$\:\:\text{y}\left(h,w\right)\in\:\left\{\text{0,1}\right\}$ be the real label, and H and W be the height and width of the image, then the cross-entropy loss can be expressed as follows:

12 $$\:{\:\:\:\:\:L}_{BCE}=-\frac{1}{H\times\:W}[y\left(h,w\right)\text{log}\left(\widehat{y}\left(h,w\right)\right)+\left(1-y\left(h,w\right)\right)\text{l}\text{o}\text{g}(1-\widehat{y}\left(h,w\right)\left)\right]\:\:\:\:\:$$

The IoU loss can be expressed as, where$\:\:\epsilon\:$ acts to prevent the divisor from being zero, which for this is taken as $\:{10}^{-6}$ experiment:

13 $$\:\:\:\:\:\:\:\:\:\:\:\:\:\:\:\:\:{L}_{IoU}=1-\frac{{\sum\:}_{h,w}\widehat{y}\left(h,w\right)y\left(h,w\right)}{{\sum\:}_{h,w}[\widehat{y}\left(h,w\right)+y\left(h,w\right)-\widehat{y}\left(h,w\right)y\left(h,w\right)]+\epsilon\:}$$

The joint loss function is (This experiment measured the highest Dice coefficient when alpha beta was set to 0.25 and 0.75.):

14 $$\:{L}_{total}=\alpha\:{L}_{BCE}+\beta\:{L}_{IoU}$$

### Evaluation indicators

Since the medical image segmentation task is essentially a classification judgment for each pixel, it can be regarded as a binary classification problem (lesion region and background) in the skin lesion segmentation problem. In order to comprehensively evaluate the segmentation performance of the proposed LMSAUnet model, the following commonly used evaluation metrics are used in this paper: Accuracy, Intersection Over Union, Dice, Frequency-weighted Intersection Over Union, and the number of trainable parameters to comprehensively measure the accuracy, robustness and its complexity of the model.

The indicators for each assessment were defined through True Positive Example (TP), False Positive Example (FP), True Negative Example (TN), and False Negative Example (FN)^39^with the following indicators:

1. Accuracy.

indicates the ratio of the number of pixels correctly categorized by the model to the total number of pixels, and is suitable for data scenarios with relatively balanced category distribution. However, in scenarios with unbalanced categories, this metric may misclassify the model performance. The calculation formula is:

15 $$\:\:\:\:\:\:\:\:\:\:\:\:\:\:\:\:\:\:\:\:\:\:\:\:\:\:\:\:\:\text{A}\text{c}\text{c}\text{u}\text{r}\text{a}\text{c}\text{y}=\frac{TP+TN}{TP+TN+FP+FN}$$

2. Intersection Over Union.

measures the degree of overlap between the predicted region and the real labeled region, which is one of the core metrics in the segmentation task, and takes the value in the range of [0, 1].The larger the IoU is, the closer the model prediction result is to the real label. The definition is as follows:

16 $$\:\:\:\:\:\:\:\:\:\:\:\:\:\:\:\:\:\:\:\:\:\:\:\:\:\:\:\text{I}\text{o}\text{U}=\frac{TP}{TP+FP+FN}\:\:$$

3. Dice coefficient.

Dice coefficient is used to measure the similarity between the predicted results and the real labeling, especially sensitive to the boundary region differences, so it is widely used in medical image segmentation tasks. Its expression is:

17 $$\:\:\:\:\:\:\:\:\:\:\:\:\:\:\:\:\:\:\:\:\:\:\:\:\:\:\:\:\text{D}\text{i}\text{c}\text{e}=\frac{2\times\:TP}{2\times\:TP+FP+FN}\:\:\:\:\:\:\:\:\:\:\:\:\:$$

4. Frequency-weighted intersection Intersection Over Union.

To solve the problem of performance evaluation bias caused by category imbalance, FWIoU introduces category frequency weighting on the basis of IoU, which can more realistically reflect the performance of the model in each category. For the dichotomous case (positive category c = 1, negative category c = 0), the formula is:

18 $$\:\text{F}\text{W}\text{I}\text{o}\text{U}=\sum\:_{c=0}^{C}\left(\frac{{N}_{c}}{{N}_{total}}\times\:Io{U}_{c}\right)$$

where$\:{N}_{c}$​ denotes the number of pixels in class c$\:,{N}_{total}\text{}$ is the total number of all pixels.

5. The number of trainable parameters.

The parameter count​​ is used to measure computational complexity and resource consumption, where fewer parameters indicate a lighter model that is more suitable for deployment in edge devices or mobile applications.

### Results on the ISIC dataset

In this paper, homology training and testing are first performed on ISIC2016, ISIC2017 and ISIC2018 datasets, where the experimental results of ISIC2016 and ISIC2017 datasets are displayed in the Appendix(A1, A2 and A4).

**Fig. 7:**
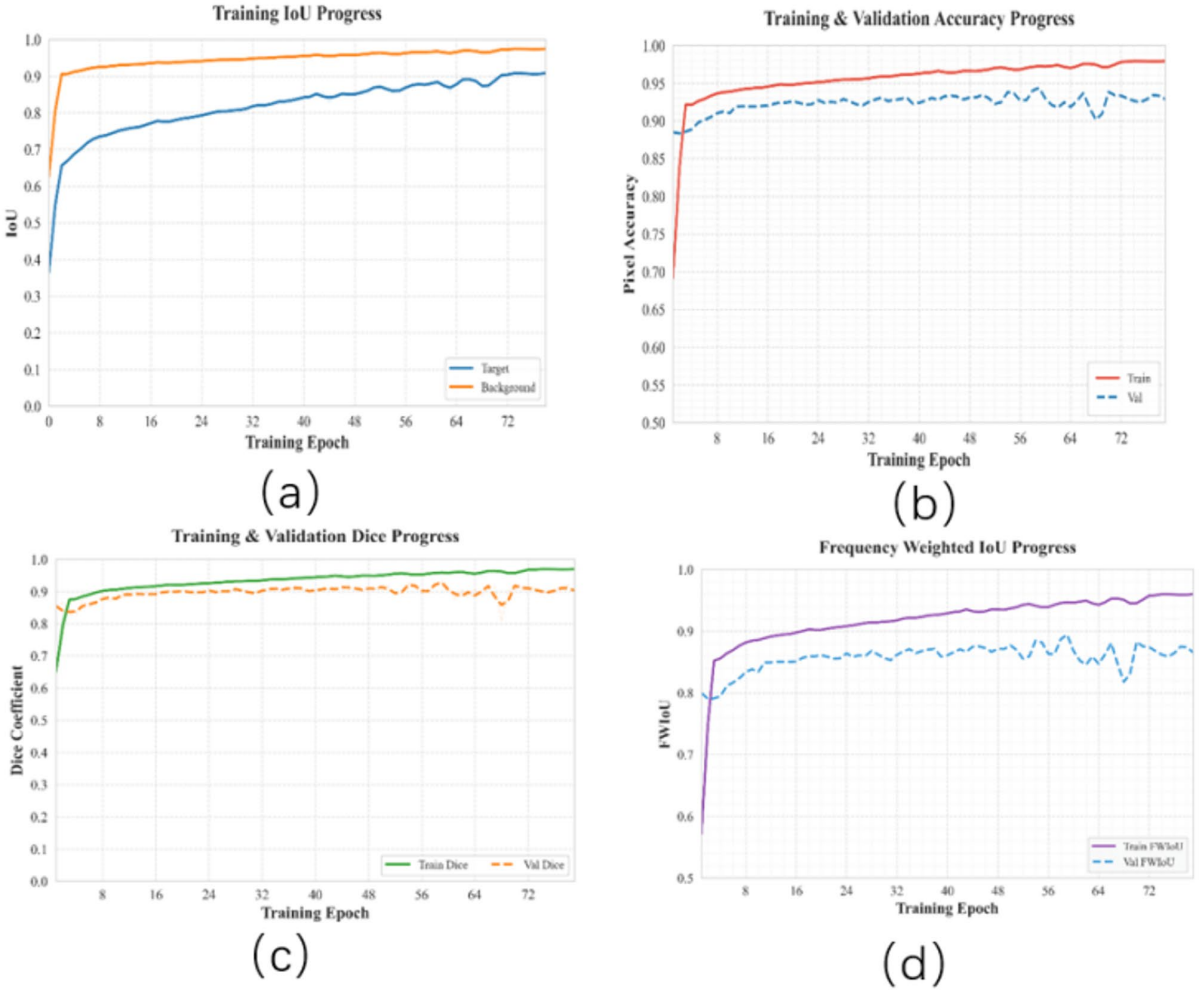
Training metrics on ISIC2018: (**a**) Target vs. Background IoU, (**b**) Training/Validation accuracy, (**c**) Dice coefficient evolution, (**d**) FWIoU progress.

The following conclusions can be drawn from Fig. 7: During the 78 full training rounds, the model exhibits significant two-stage optimization features (Fig. 7 Section a): the first is a rapid ramp-up period in the first eight rounds: the background IoU from an initial 0.7 jumps to more than 0.92, and the target region IoU from 0.4 increases to 0.75, demonstrating the model’s ability to quickly capture low-level visual features early on. In the remaining rounds, the background IoU increases slowly to 0. 9741 at a rate of 0.002/round, and the target region IoU reaches 0.9077 at a rate of 0.003/round, which is consistent with the late optimization property of gradient descent. The final difference of between the two types of IoU 0.0664 reflects the inherent challenge of lesion edge segmentation.

The comparative analysis of the metrics between the training set and validation set reveals the following findings: 1. From (Fig. 7 Section b) the difference between the pixel accuracy of the training set (0.9794) and the validation set (0.9265) is maintained at 5.29%, which is lower than the common generalization gap for medical image segmentation tasks (usually > 8%), proving the accuracy of in pixel classificationthe LMSAUnet. the Dice coefficient curve (Fig. 7 Section c) shows that the validation set enters stability after 60 rounds (0.8992 ± 0.004), indicating excellent model resistance to overfitting.The final value of FWIoU (Fig. 7 Section d) for the validation set (0.8611) is 10.3% lower than that of the training set (0.9599), and the difference is positively correlated with the percentage of lesion area (12.7% on average for ISIC2018), which is consistent with the mathematical frequency-weighted indicator Characterization.

Due to the use of cosine annealing learning rate, after checking the learning rate reaches the maximum in 64 to 72 rounds, resulting in fluctuations in various indicators of the model, the follow-up can be optimized learning rate algorithm to make the model convergence more stable and avoid the local optimal situation.

**Table 3:** Quantitative comparison of segmentation models showing top-performing (bold) and runner-up (italic) metrics. Our method achieves 3 best and 1 s-best results.

Model	Unet	DeeplabV3	TransUnet	SegNet	BiseNet	UNeXt	EGE_UNet	UCM_Net	MUCM_Net	Ours
Dice	0.8678±0.010	*0.8779±0.008*	0.8256±0.011	0.8427±0.008	0.8436±0.010	0.8423±0.010	0.8365±0.010	0.8506±0.011	0.8497±0.010	**0.8792±0.011**
MIoU	0.8346±0.010	*0.8450±0.010*	0.7893±0.012	0.8146±0.011	0.8409±0.011	0.8276±0.011	0.7989±0.011	0.8163±0.010	0.8214±0.010	**0.8462±0.009**
FWIoU	0.8570±0.009	0.8427±0.010	0.8272±0.009	0.8460±0.010	**0.8896±0.010**	0.8583±0.011	0.8341±0.009	0.8499±0.012	0.8524±0.010	*0.8755±0.010*
Accuracy	0.912±0.0110	0.9081±0.010	0.9038±0.010	0.9142±0.010	0.9091±0.010	*0.9223±0.010*	0.8277±0.010	0.9177±0.010	0.9184±0.010	**0.9262±0.010**

**Table 4:** This table compares the parameters (M) and flops (G) for different segmentation models, including unet, DeeplabV3, transunet, segnet, bisenet, unext, ege_unet, ucm_net, mucm_net and the proposed model (marked in bold for the best, italic for second-best).

Model	Unet	DeeplabV3	TransUNet	SegNet	BiseNet	UNeXt	EGE_UNet	UCM_NET	MUCM_Net	Ours
Params(M)	37.66±3.0	39.04$\:\pm\:2$.0	66.82$\:\pm\:5$.0	29.44$\:\pm\:2$.0	2.92$\:\pm\:0$.3	1.93$\:\pm\:2$.0	**0.046** $\:\pm\:$ **0.002**	*0.047* $\:\pm\:$ *0.003*	0.081$\:\pm\:$0.005	0.39$\:\pm\:$0.002
Flops (G)	52.06±1.0	9.20$\:\pm\:1$.0	25.00$\:\pm\:$1.0	30.75$\:\pm\:2$.2	1.67$\:\pm\:$1.5	8.03$\:\pm\:1$.0	*0.072* $\:\pm\:$ *0.003*	**0.054** $\:\pm\:$ **0.005**	0.073$\:\pm\:$0.004	1.06$\:\pm\:$0.100

### Comparative analysis of model performance test setbased on ISIC 2018

Tables 3 and 4 present the experimental results of different models on the ISIC2018 dataset. To ensure a fair and authoritative comparison, we selected several classic segmentation models as baselines, which have been widely adopted and validated (see Supplementary Information A5 for details). Therefore, we set the upper limit for the number of parameters to 5 M, selected four non-lightweight segmentation models and five lightweight segmentation models, and chose the model with the smallest loss value from the validation set to apply it to the corresponding test set for testing, thereby obtaining the above data and comparative analysis. The experimental results show that the LMSAUnet model performs best on this dataset, achieving the highest Dice coefficient, mean intersection over union (MIoU), and accuracy. Compared to other models, LMSAUnet outperforms the second-place model (DeeplabV3) by 0.0013 in Dice coefficient, 0.012 in MIoU, and 0.0141 in accuracy. In terms of FWIoU, it ranked second, just behind BiseNet.

Overall, it can be inferred that larger and deeper models are not necessarily better. The selected lightweight models have achieved performance comparable to large-parameter models, and the method proposed in this paper demonstrates competitive advantages in terms of accuracy and lightweight design. Although the three lightweight models have fewer parameters and computational requirements, they still lag behind large-parameter models in terms of performance, yet they also highlight the potential of lightweight models. LMSAUnet has only 0.39 million parameters, less than 1.04% of U-Net, and compared to the lightest EGEUnet (0.046 million), it achieves improved performance with slightly more parameters. Moreover, the 0.39 million parameters do not impose a computational burden. This superiority can be attributed to three innovative designs (see method for details):

1. Cross-scale feature distillation module captures multi-scale information.
2. Separable convolutions replace traditional convolutions, significantly reducing the number of parameters without sacrificing the receptive field.
3. Channel-spatial attention mechanism complements the insufficient interactivity of separable convolutions.

**Table 5:** Comparative segmentation results on the PH2 dataset from three models (UNet, DeeplabV3, TransUNet) trained on ISIC2018, demonstrating cross- dataset generalization capabilities.

Model	Accuracy	Dice	MIoU
LMSAUnet	0. 9164	0. 8649	0. 8421
TransUnet	0. 8896	0. 8196	0. 8299
DeeplabV3	0. 8938	0. 8621	0. 8116
EGE_UNet	0.9032	0.8602	0.8386
UCM_Net	0.8919	0.8513	0.8227

### External dataset results

To evaluate the generalization ability of the proposed model on untrained datasets, we designed a cross-dataset experimental scheme: the model was trained on the ISIC2018 dataset and tested on the PH² dataset. The experimental results are shown in Table 5. Our method achieved the best performance in terms of accuracy, Dice, and average IoU, and there was no significant performance degradation compared to the training set. However, other generalization capabilities did not meet expectations, further validating the significant advantages of the proposed lightweight network in cross-domain generalization tasks.

### Ablation experiments

Ablation studies are necessary to understand the contribution of components in the model to the model, and to further demonstrate the lightweight and feature extraction capabilities of ECDF, tests were conducted in SegNet, and TransUnet using the ECDF module, and the modules for extracting features in SegNet and TransNet were directly changed to ECDF modules.

**Table 6:** Performance comparison between baseline and ECDF-enhanced architectures, demonstrating the effectiveness of replacing conventional feature extraction modules with our proposed ECDF block.

Model	Use block	Params (M)	FLOPS (G)	Dice (ISIC2018)
SegNet	Original ECDF	29. 44 0.620	30.75 6.980	0. 8427 0. 8496
TransUnet	Original ECDF	66.82 38.40	25.00 13.32	0. 8256 0. 8241

As shown in Table 6, with the parameter amount reduced by about 47 times and FLOPs reduced by about 4.4 times, the Dice coefficient of SegNet^40^ improves by 0. 0069 compared to the original model, demonstrating the potential for some performance gain even at very low cost.

In addition, although TransUNet consumes a large amount of parameter and computational resources due to the introduction of the attention mechanism, it shows little significant performance degradation under the condition that the number of parameters and computational effort are halved. We attribute this result to the proposed ECDF module, which effectively exploits the redundant information between channels while maintaining a lightweight design, acquires multi-level sensory field features with low computational overhead, and further enhances the feature representation capability by combining the space-channel attention mechanism.

Experimental results show that the ECDF module can be flexibly integrated into various mainstream network architectures by simply adjusting the number of channels, which significantly improves its performance in resource-constrained scenarios.

**Table 7:** This table presents a comparative analysis of model performance with and without the Spatial- channel attention (SCA) mechanism on the ISIC2018 dataset, highlighting the quantitative improvements in segmentation accuracy.

Model	Accuracy	Dice	MIoU
No SCA	0. 8963	0. 8548	0. 8399
EXIST SCA	0.9262	0. 8792	0. 8462

As shown in Table 7,the experimental results show that the introduction of the spatial channel attention (SCA) module significantly improves the model performance: where Accuracy​​ improves by 0.0299,Dice​ improves by 0.0244,and MIoU improves 0. 0063. The spatial channel attention mechanism is verified to be effective for feature capture in the segmentation task by dynamically fusing spatial and channel features.

**Table 8:** This table presents the impact of different scaling factors on model parameters, computational complexity, and performance metrics.

Compression ratio	Params (M)	FLOPS (G)	mIoU
2	0.83	2.23	0. 8823
4	0.59	1.61	0. 8799
8	0.39	1.30	0. 8792
16	0.32	1.15	0. 8719
32	0.29	1.07	0. 8653

In order to verify the effect of channel compression factor N on model performance and efficiency, this paper sets $\:\text{N}\in\:\{\text{2,4},\text{8,16,32}\}$ on ISIC 2018 dataset for experiments respectively. The experimental results are shown in Table 8 With the increase of N, the number of model parameters and the amount of inference computation decrease significantly, but too large a compression ratio will bring the problem of feature expression ability decreasing, which ultimately leads to the decrease of segmentation performance. Considering the model performance and efficiency, *N* = 8 is chosen as the default setting in this paper.

**Fig. 8:**
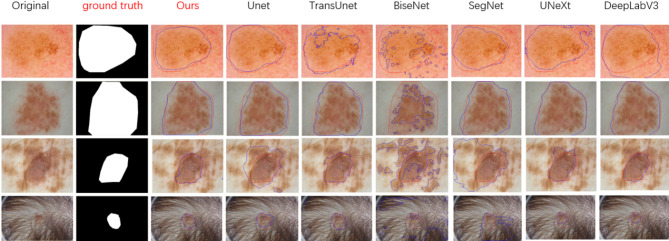
This figure displays the original image, ground truth annotation, and segmentation results from various models. The red contour represents the ground truth region, while the blue contour indicates the predicted region by the model.

## Discussion

The LMSAUnet proposed in this study is a based on the depth-separable convolution skin lesion segmentation model mechanism of and attention, and its main component, the ECDF module, dramatically reduces the number of parameters and computation, while fusing multiple scales of information, and finally, through the through deep convolution and point-by-point convolutionspatial channel attention, the model pays attention to to the channels and regions that have a high contribution, and suppresses the interference of irrelevant regions, to Reducing the influence of external factors the tasksegmentation enables LMSAUnet to maximize the learning of feature distribution information in a limited dataset, and in order to enhance the model’s focus on the overall segmentation effect rather than just the classification accuracy of a single pixel, we adopt a composite loss function combining IoU (intersection and concurrency ratio) and cross-entropy.

The results show that LMSAUnet has a stronger feature extraction capability, and in the ISIC2018 datasetwhile maintaining lightnessaccuracy, Dice coefficient, and MioU achieve the highest values(Performance on the ISIC2017 and ISIC2016 datasets is shown in Appendix A3.). To more intuitively demonstrate the segmentation effects of different models, we conducted a visualization analysis.As can be seen from Fig. 8, in complex environments, TransUnet, BiseNet, and SegNet cause segmentation errors due to hair and skin colorcoefficient and MioU achieve the highest values., while DeepLabV3, although excellent from the data, can be seen from the graph that there are still the edge blurring and loss of detailed features due to the convolution of voids, and UneXt is too sensitive the edgesa good performance, but to, which results in over-segmentation, and overall, Unet has Dice Unet has a good performance, but compared to our proposed it is still poor model, and LMSAUnet best accomplishes the segmentation task while maintaining the lightest weight.

However, there is still room for improvement in this study as follows: first, slight segmentation bias still occurs at very small lesion protrusions with an average error of about 2 pixels, which may be related to the loss of high-frequency details due to continuous downsampling operations in the current network architecture. Second, although the model has met the practical requirements in terms of inference speed and cross-domain adaptability, it has not yet been subjected to rigorous clinical validation centers, especially the lack of evaluation involving specialized physicians. Future work will focus on the following improvements: (1) mitigating the information loss due to downsampling by introducing a learnable sub-pixel convolutional layer; (2) conducting prospective clinical studies in collaboration with tertiary hospitals to collect data from at least 500 pathologically-confirmed cases for model validation; and (3) developing a lightweight version for real-world clinical deployments, and optimizing the model to enable it to be used on mobile medical devices (e.g., portable dermoscopes) (e.g., portable dermoscopes) in real time. These improvements will significantly increase the utility of the model in real-world medical scenarios.

## Conclusion

In this paper, a novel lightweight skin lesion segmentation model, LMSAUnet, is proposed, aiming at solving the challenges brought by factors such as illumination, equipment differences, and skin color changes, which are common in practical applications. Since there are convenience and validity problems in the application of oversized models in real clinics, and traditional methods are prone to over-segmentation, under-segmentation, mis-segmentation, and detail loss, how to improve the segmentation accuracy and generalization ability has become a key issue. In order to cope with these problems, we propose a new model that employs an ECDF module with depth-separable convolution and attention mechanism by removing the convolutional part in U Net for the redundant channel characteristics in neural networks. This module is not only able to fuse the information from multiple receptive fields into one layer, which significantly improves the feature extraction capability, but also greatly reduces the number of parameters and computational complexity of the model.

Experimental results on multiple datasets show that LMSAUnet excels in metrics such as accuracy, Dice coefficient and M IoU, while greatly reducing the computational cost of the model. Due to the low coupling between LMSAUnet and ECDF module, ECDF module can be flexibly integrated into other mainstream network architectures, thus effectively reducing the number of parameters and improving the generalization ability of the model, making it feasible for practical applications. Our study fully demonstrates the potential of LMSAUnet in the task of skin lesion segmentation under resource-constrained conditions, especially in variable clinical environments, to provide accurate segmentation results in a stable and efficient manner.

Although our model performed well in multiple experiments, there are still some shortcomings. Future work could consider combining more multimodal information or further optimizing the ECDF module to improve segmentation performance in more complex scenarios, and further scientifically evaluate the model’s capabilities using clinical results.In addition, as the size of the dataset increases, how to further improve the training efficiency and inference speed of the model on large-scale data is still a research direction worth exploring.

Overall, the LMSAUnet proposed in this study provides a new solution for the task of skin lesion segmentation and shows great potential for application with limited resources. With the advancement of technology, we believe that this method will play an important role in real-world medical image analysis.

## Supplementary Information

Below is the link to the electronic supplementary material.

Supplementary Material 1

## Data Availability

The datasets used in this study are publicly available at https://challenge.isic-archive.com/data/The code for this study is available at https://github.com/hubosheng123/skin-disease.
