# Supplementary material for: Lightweight Unet with depthwise separable convolution for skin lesion segmentation
Source: Sci Rep. 2025 Oct 2;15:34327. doi: 10.1038/s41598-025-16683-1 (PMC12491560; doi:10.1038/s41598-025-16683-1)
Supplement: Supplementary file 1 — Supplementary Material 1 [file 41598_2025_16683_MOESM1_ESM.pdf]

Lightweight Unet with Depthwise Separable Convolution for Skin Lesion Segmentation

Yong Li[1],Bosheng Hu[1],Menghao Song[1], Kun Liu[1]

[1]College of Computer Science and Engineering, Northwest Normal University, Lanzhou 730070, China

Corresponding Author:

Yong Li*

Northwest Normal University, Lanzhou 730070, China.
E-mail: facingworld@nwnu.edu.cn

Supplementary experimental results

This appendix provides additional experimental results not shown in detail in the main text. Included are the training and validation process metrics curves for models on the ISIC 2016 and ISIC 2017 datasets.

A.1 Training curves on the ISIC 2016 dataset

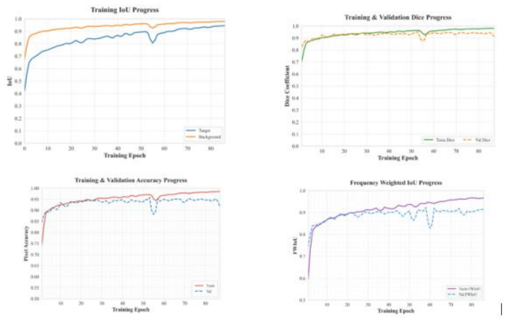

The trend of each performance metric (IoU, Dice coefficient, Accuracy, FWIoU) during training is shown on the ISIC 2016 dataset.

A.2 Training curves on the ISIC 2017 dataset

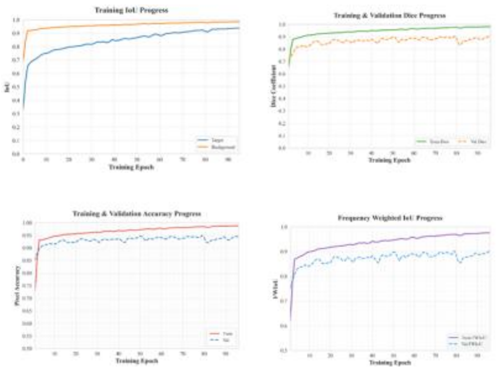

The trend of each performance metric (IoU, Dice coefficient, Accuracy, FWIoU) during the training process on the ISIC 2017 dataset is shown.

A.3 LMSAUnet Performance in ISIC 2016 and 2017 Test Sets

Year	Dice	Accuracy	MIoU	FWIoU
2016	0.8743	0.9134	0.8479	0.8697
2017	0.8764	0.9158	0.8424	0.8731

A.4 Other notes

- All training curves are plotted based on model weights saved every 5 epochs.
- The evaluation metrics are consistent with those in the main text, ensuring consistency and comparability of experiments.

A. 5 Comparative model description

1. U-Net(Ronneberger et al., 2015),Landmark work in medical image segmentation, symmetric encoder-decoder architecture, introduction of jump connections to mitigate information loss

2.DeeplabV3(Chen et al., 2017),a representative method for semantic segmentation of natural images, uses empty space pyramid pooling (ASPP) to capture multi-scale context, which is computationally efficient but sensitive to small target segmentation.

3. BiseNet (Yu et al., 2018), lightweight representative model, lightweight dual-path architecture for real-time scenarios, retaining details through spatial paths and extracting semantics through contextual paths, very low number of parameters, but limited accuracy

4. UNeXt(Valanarasu et al., 2022), a lightweight hybrid design of CNN and Transformer that achieves SOTA efficiency-accuracy balance at 1-2M parameter scales for mobile deployment.

5.TransUNet (Chen et al., 2021), the first hybrid architecture to introduce Transformer into medical image segmentation, outperforms pure CNNs in long-range modeling, but has a large number of parameters and high computational cost.

6.SegNet(Badrinarayanan et al., 2015) Representative work on early encoder-decoder architecture with low memory footprint by pooling indexes.

7.EGEUnet(Ruan *et al.,2023*) is a medical image segmentation network that combines global and local features, an efficient self-attention mechanism, and improved skip connections to optimize computational efficiency while maintaining accuracy.

8.MUCM-Net (Yuan *et al.* ,2024)aims to enhance the model's ability to model key features when processing complex visual tasks by introducing a dual attention mechanism, significantly improving performance in tasks such as image segmentation and object detection.

9.The UCM network(Yuan *et al.,2024*) effectively improves performance in medical image segmentation tasks by combining local and global information in a dual-path structure, particularly when dealing with complex and blurred boundaries.
